# Culture of spermatogonial stem cells and use of surrogate sires as a breeding technology to propagate superior genetics in livestock production: A systematic review

**DOI:** 10.14202/vetworld.2021.3235-3248

**Published:** 2021-12-31

**Authors:** Wilkister Nakami, Ambrose Ng’eno Kipyegon, James Nguhiu-Mwangi, Christian Tiambo, Stephen Kemp

**Affiliations:** 1 Department of Clinical Studies, Faculty of Veterinary Medicine, University of Nairobi, 29053-00625 Nairobi, Kenya; 2 Livestock Genetics Program International Livestock Research Institute, 30709-00100, Nairobi, Kenya; 3 Centre for Tropical Livestock Genetics and Health (CTLGH)-ILRI, 30709-00100, Nairobi, Kenya

**Keywords:** culture, donor-derived spermatogenesis, marker, spermatogonial stem cells, transfection, transplantation

## Abstract

**Background and Aim:**

Spermatogonial stem cells (SSCs) have previously been isolated from animals’ testes, cultured *in vitro*, and successfully transplanted into compatible recipients. The SSC unique characteristic has potential for exploitation as a reproductive tool and this can be achieved through SSC intratesticular transplantation to surrogate sires. Here, we aimed at comprehensively analyzing published data on *in vitro* maintenance of SSC isolated from the testes of livestock animals and their applications.

**Materials and Methods:**

The literature search was performed in PubMed, Science Direct, and Google Scholar electronic databases. Data screening was conducted using Rayyan Intelligent Systematic Review software (https://www.rayyan.ai/). Duplicate papers were excluded from the study. Abstracts were read and relevant full papers were reviewed for data extraction.

**Results:**

From a total of 4786 full papers screened, data were extracted from 93 relevant papers. Of these, eight papers reported on long-term culture conditions (>1 month) for SSC in different livestock species, 22 papers on short-term cultures (5-15 days), 10 papers on transfection protocols, 18 papers on transplantation using different methods of preparation of livestock recipients, and five papers on donor-derived spermatogenesis.

**Conclusion:**

Optimization of SSC long-term culture systems has renewed the possibilities of utilization of these cells in gene-editing technologies to develop transgenic animals. Further, the development of genetically deficient recipients in the endogenous germline layer lends to a future possibility for the utilization of germ cell transplantation in livestock systems.

## Introduction

Spermatogenesis is the process through which spermatozoa are produced in males. The process is highly specialized and is dependent on the continuous actions of spermatogonial stem cells (SSCs). An equilibrium between self-renewal of SSC and the production of differentiating spermatogonia is key to sustaining optimal sperm production while preventing exhaustion of the stem cell reservoir. Regulation of SSC fate is partly influenced by signaling from growth factors which are synthesized by the somatic stem cell niche support cells, most importantly Sertoli cells [1,2]. SSCs have a unique potential to expand *in vitro* and form colonies of undifferentiated spermatogonia. These *in vitro* cultured SSCs when transplanted to testes of live recipient animals reestablish spermatogenesis producing sperms of donor-derived haplotype [3]. The use of SSC to form gametes from specific sires provides an opportunity for genetic improvement in livestock. However, successful transplantation of SSC requires the establishment of robust and effective *in vitro* culture systems. Such a system will ensure that the small number of SSC (0.03% of total testicular cells) isolated from the testes can be expanded to millions before transplantation [1]. Lack of methodologies for long-term expansion of SSC in culture and effective methods for the preparation of ideal recipients for transplantation of donor SSC has limited exploitation of SSC transplantation as an alternative breeding technology in livestock production systems [3]. Limited studies have documented protocols for long-term expansion of SSC in livestock species with varying success [3-6]. The ultimate proof of the existence of SSC in a culture dish is through transplantation and reestablishment of donor-derived spermatogenesis in the testes of the recipient animal [7]. In addition, successful culture systems of livestock species SSC will herald opportunities to study gene functions and explore gene editing methodologies for the *in vitro* cultured SSC.

Here, we aimed at collating data on *in vitro* culture systems of spermatogonial stems cells in livestock, specific SSC markers for identification, and gene manipulation of these cells. Thereafter, to document the standardized, workable, and reproducible *in vitro* culture conditions and feasible applications of SSC in livestock production systems.

## Materials and Methods

### Ethical approval

No experiments were conducted on live animals in this study, so ethical approval was unnecessary.

### Data sources and search strategy

In line with Preferred Reporting Items for Systematic reviews and Meta-Analyses guidelines [8], a systematic literature search was performed. Searches were conducted in multiple electronic databases: PubMed, ScienceDirect, and Google Scholar for research articles published between January 1990 and February 2021. An initial and subsequent keyword searches with various combinations of search terms such as SSC terminologies, culture, and livestock species were undertaken. Keywords and subject terms included: (“spermatogonial stem cells”) OR (“undifferentiated spermatogonia”) OR (“male germline stem cells”) OR (“spermatogonial stem cell transplantation”) OR (“donor-derived spermatogenesis” OR (“spermatogonial stem cell transfection”) AND (“culture”) AND (“livestock”) OR (“cattle”) OR (“SHEEP”) OR (“goats”) OR (“Bovine”) OR (“Pigs”) OR (“camels”).

### Selection criteria and data extraction

Articles were included if they comprised original research published in a peer-reviewed journal and reported on the culture of spermatogonia cells in at least one livestock species. We excluded articles if; (i) articles were in non-English language, (ii) studies of spermatogonia stem cells in were non-livestock species, (iii) abstracts were not published as a full manuscript, and (iv) non-experimental studies. Article searches and screening were performed by considering article titles and abstracts for inclusion according to the search criteria. Data extraction from studies was performed by one author (WNN) and independently checked by another author (ANK) using a customized checklist. All the articles from the three electronic databases were exported to the Mendeley reference manager. Duplicate articles were excluded and the resultant data file from each of the databases was exported to Rayyan systematic reviews software (https://www.rayyan.ai/) [9] for screening.

### Statistical analysis

For all the included studies, we categorized SSC *in vitro* culture systems into the following groupings: (i) Studies on *in vitro* culture of SSC for short-term ≤21 days or long-term culture ≥21 days; (ii) studies on SSC characterization using specific SSC and general pluripotent markers; (iii) studies on SSC transplantation, methods of recipient preparation, and fate of donor SSC; and (iv) studies on SSC gene manipulation methodologies. The quality of articles included in the review was accessed using the Cochrane handbook for systematic reviews version 6.2. Cochrane, 2021 (Available from www.training.cochrane.org/handbook). Each article was evaluated based on methodological study design and grouped according to the following categories: (i) Good quality studies; methodology as clear and precise mainly on multiparameter enrichment procedures for SSC, confirmation of SSC markers through real-time polymerase chain reaction and immunochemistry and SSC transplantation and (ii) medium quality: Clear methodology on isolation, enrichment, and characterization of SSC through immunostaining only.

## Results

### Description of included studies

**Figure-1:**
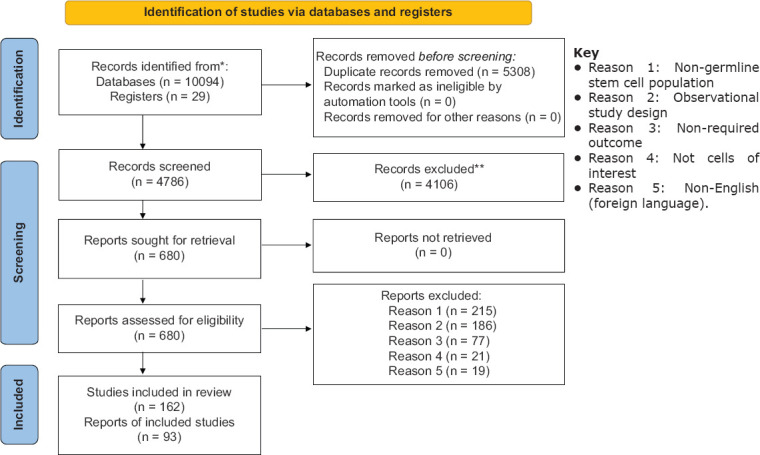
Preferred reporting items for systematic reviews and meta-analyses (PRISMA) literature search strategy [8].

Of the 4786 articles retrieved, 162 studies were reviewed (Figure-1); and 93 studies met all inclusion criteria. The 93 studies were geographically diverse and included 13 countries. Geographical distribution was follows: Iran n=19, the USA n=17, China n=15, Australia n=14, Korea n=6, Japan n=1, the United Arab Emirates n=1, India n=6, Brazil n=3, Canada n=4, the Netherlands n=5, Finland n=1, and Switzerland n=1. There was no study associated with SSCs in any species in Africa. Thirty-six studies focused on *in vitro* culture of SSC, 21 studies on identification of specific markers of SSC, 23 studies on transplantation of SSC, six studies on transfection, and five studies on donor-derived spermatogenesis. Categorization of studies based on the livestock species in which the study was conducted was as follows: Bovine n=36, goats n=23, pigs n=17, sheep n=16, and camels n=1. Record on the year of publication of the studies was as follows: the Year 2016-2021, n=28 studies; the year 2005-2015, n=50; and year 1990-2004, n=14. The *in vitro* culture of SSC from livestock animals was first published in 1999. The early studies focused mainly on the isolation of a mixed germ cell population, including SSC and short-term culture of the cells [10-13]. Since then, there have been striking advances in the standardization of protocols for isolation, purification, characterization, and culture of SSC. Furthermore, the SSC transplantation technology has been explored for its viability in the production and dissemination of superior male gametes in livestock production systems. The synthesis of the data was qualitative and can be found in the subheadings below.

### Long-term culture of SSC and the *in vitro* culture conditions required

The duration of which SSCs were maintained in culture was categorized into three: Culture for 1-3 days (n=8), culture 5-18 days n=23, and >21 days n=7. Of much interest was the long-term culture of the SSC for a period of >21 days (Table-1) [3-6,11,14-16]. From the review findings, the longest period of SSC culture was three months on a feeder cell monolayer (Sandos inbred mouse [SIM]-derived 6-thioguanine- and ouabain-resistant cells [STO]) using a serum-free medium (Knockout serum replacement) [6]. The SSC from immature and mature bovine testis stably expressed SSC markers during the culture period. Interestingly, SSC from mature testis required supplementation with drug 6-bromoindirubin-3′-oxime (BIO) in culture. The drug BIO activates the signaling pathway involved in the maintenance of undifferentiated spermatogonia from adult testes during the early stage of *in vitro* culture. In addition, bovine SSCs from an immature testis were maintained on serum-free medium on bovine fetal fibroblast feeder cells for 2 months [3]. However, when the bovine SSCs were cultured on laminin-coated plates (feeder-free) in preconditioned serum-free medium, the cells could only persist in culture for one month. These SSCs in culture expressed SSC-specific markers in cattle promyelocytic leukemia zinc finger (*PLZF*)*/ZBTB16* and *LIN28* [3]. Finally, bovine SSCs were also cultured in serum-free medium on bovine somatic testicular cells feeder layer for 3-12 weeks [5]. In general, from the findings of the review, long-term cultures of bovine SSC utilized serum-free medium on feeder cell layer with a cocktail of growth factors which included: Glial cell line-derived neurotrophic factor (GDNF), fibroblast growth factor 2 (FGF2), colony-stimulating factor-1, and stromal cell-derived factor (SDF-1). The long-term bovine SSC culture studies reported the detrimental effects of serum on SSC self-renewal and thus used a serum-free medium [3,5,6]. On the contrary, in sheep and goat, the long-term culture medium of SSC was supplemented with fetal bovine serum (FBS) [14,15]. The proliferation of SSC in culture was reported for extended periods although the evidence of SSC undifferentiated status through marker expression cells expressed *CDH1*, *UCHL1*, *GFRα1*, *PLZF*, and *ITGA6* in sheep and of *PLZF*, *α6 integrin* in goats was not sufficient to conclude that the cultures were indeed made up of undifferentiated SSC.

**Table 1:** Summary of studies with reports on the long-term culture of SSC in livestock species.

Culture period	Species	Age of donor	Culture period	Growth factors	Medium used	Markers evaluation	Culture conditions
Suyatno *et al*. [6]	Bovine	3 months	3 months	LIF or GDNF	15% KSR and 1% FBS for 5 days n then 20% KSR	Dome-shaped ES cell-like colonies UCHL-1, *DBA*. PCR detection: NANOG, OCT4, SOX2	3 months in SFM in 5% CO2 37°C STO feeder cells
Oatley *et al*. [3]	Bovine	4-5 months	2 months	GDNF, FGF, LIF	StemPro serum-free	Germ cell clumps *PLZF*, *LIN28*, GFRA1, ID4, *NANOS2* markers	5% CO_2_, 10% O_2_ at 35°C on bovine fetal fibroblast feeder for 2 months 1 month on laminin-coated plates n preconditioned media
Crouse, [5]	Bovine	3-4 months	3 weeks	GDNF, FGF, SCF SDF	StemPro serum-free	*PLZF*	3-week BSC feeder layer better than BEF 37°C
Pramod *et al*. [14]	Goat	3-4 months	2 months	NONE	10% FBS on Sertoli cell layer	*PLZF*, a6 integrins	Sertoli feeder layer 37°C, 5% CO_2_
Binsila *et al*. [15]	Sheep	Prepubertal rams	36 days	GDNF, IGF, EGF	StemPro, 10% FBS	*PLZF*, ITGA, *GFRα1*	Laminin-coated plates feeder-free culture
Izadyar *et al*. [16]	Bovine	5 months	3 months	none	2.5% FCS in MEM	*DBA*, colonies, cells differentiated into spermatids	Sertoli feeder layer 5% CO2
Dobrinski *et al*. [11]	Boar, bovine	6-week boar, 6-month bovine	1 month	none	DMEM	None. Cells transplanted to mice testes	STO feeder 32°C, 5% CO_2_
Aponte *et al*. [4]	Bovine	4-6 months	25 days	None	MEM with 2.5% FCS and fungizone	Blob-like colonies Cells transplanted to mice testes	A monolayer of Sertoli cell developed in the germ cell culture

KSR=Knockout serum replacement, SFM=Serum-free media, DMEM=Dulbecco’s Modified Eagle Medium, MEM=Minimum essential media, FBS=Fetal bovine serum, FCS=Fetal calf serum, STO=Sandos inbred mouse (SIM)-derived 6-thioguanine- and ouabain-resistant (STO) cells, SDF=Stromal cell-derived factor, SCF=Stem cell factor, BEF=Bovine embryonic fibroblast

### Characterization of SSC in culture using specific SSC markers in livestock

Markers used for SSC characterization in the review included: *VASA, PLZF, THYI* (*CD9*), Ubiquitin carboxy-terminal hydrolase 1 (*UCH-LI*) protein gene product 9.5 (*PGP9.5*), *αIntegrins*, Dolichosbiflorus agglutinin (*DBA*), *OCT 4*, *GFRα1*, *LIN28*, and *NANOG* (Table-2) [3-6,12,14,15,17-71]. In all studies (n=74), where SSCs were cultured, expression of more than one marker in SSC was evaluated for their identification, in addition to the typical morphology of germ cell colonies. *UCH-L1*, also called *PGP9.5* was the most commonly used marker (n=47). Expression of *PLZF* transcription factor was evaluated in 21 studies and was also the main specific marker used for identification of bovine, caprine, and porcine SSC. Thymocyte differentiation antigen 1 (*THY1*) expression was reported in SSC in 15 studies. Evaluation of SSC expression of the *LIN28* gene was conducted in only one study and was suggested to be uniquely expressed by SSC [3]. From the review, different studies used different markers for the verification of SSC undifferentiated status; however, expression of *PLZF* as being confined to SSC only not only rodents but also livestock has been supported by recent studies in bovine [3,5], sheep [17,18], pig [19], and goat [20,21].

**Table 2:** Summary of markers used in SSC characterization in livestock species.

Markers	Bovine	Ovine	Porcine	Caprine	Camel	Total
*VASA*	Oatley *et al*.[3], Kim *et al*. [22], McMillan *et al*. [31] n=3	Borjigin *et al*. [32], Borjigin *et al*. [17], Herrid *et al*. [28] n=4	Kim *et al*. [19], Zhang *et al*. [33] n=2	Wu *et al*. [34], Bahadorani *et al*. [29], Wang *et al*. [35], Niu *et al*. [36] n=4	0	n=12
*PLZF*	Oatley *et al*. [3], Reding *et al*. [37], Crouse *et al*. [5], Cai *et al*. [38], Anglin *et al*. [39], McMillan *et al*. [31] n=6	Borjigin *et al*. [32], Borjigin *et al*. [17] n=2	Lee *et al*. [40], Lee *et al*. [41], Lee *et al*. Kim *et al*. [19], Zhang *et al*. [33] n=5	Abbasi *et al*. [42], Pramod *et al*. [14], Ren *et al*. [43], Zhu *et al*. [44], Bahadorani *et al*. [29], Sharma *et al*. [21], Abbasi *et al*. [23], Song *et al*. [20] n=8	0	n=21
*THY 1*	Tajik *et al*. [30], Giassetti *et al*. [45], Reding *et al*. [37], Nasiri *et al*. [46], Youssefi *et al*. [47] n=5	Binsila *et al*. [18] n=1	Not reported	Abbasi *et al*. [42], Wu *et al*. [34], Ren *et al*. [43] Bahadorani *et al*. [29], Kaul *et al*. [26], Sharma *et al*. [21], Abbasi *et al*. [23], Song *et al*. [20] n=8	0	n=14
*UCHL1 (PGP9.5)*	Suyatno *et al*. [6], Giassetti *et al*. [48], McMillan *et al*. [31]. Kim *et al*. [49], De Barros *et al*. [50], Herrid *et al*. [27], Kim *et al*. [22], Herrid *et al*. [51], Redden *et al*. [52] n=9	Binsila *et al*. [15], Binsila *et al*. [18], Pan *et al*. [53], Moghaddam *et al*. [54], Zandi *et al*. [55], Rodriguez-Sosa *et al*. [56], Borjigin *et al*. [17], Herrid *et al*. [28] n=8	Lin *et al*. [25], Luo *et al*. [57], Lee *et al*. [40], Kim *et al*. [24], Luo *et al*. [58], Kim *et al*. [19] n=8	Sharma *et al*. [21] Wang *et al*. [35] Song *et al*. [20] Zeng *et al.* [75] Mohammad *et al*. [59] Heidari *et al*. [60], Shirazi *et al*. [61], Heidari *et al*.[62] n=8	0	n=33
*OCT 4*	Tajik *et al*. [30], Nasiri *et al*. 2012 [46], Shafiei *et al*. [63], Jabarpour and Tajik,[64] n=4	Qasemi-Panahi *et al*.[65] n=1	Not reported	Wang *et al*.[35] n=1	0	n=6
*DBA*	Suyatno *et al*. [6], Herrid *et al*. [66], Izadyar *et al*. [12], Aponte *et al*. [4], Kim *et al*. [22], Herrid *et al*. [51], Redden *et al*. [52], n=7	Not reported	Zhang *et al*.[33] n=1	Bahadorani *et al*. [29], Sharma *et al*. [21], Song *et al*.[20] n=3	0	n=11
*Gfr1*	Suyatno *et al*. [6], Kim *et al*. [49], De Barros *et al*. [50], Oatley *et al*. n=4	Rasouli *et al*. [67], Binsila *et al*.[15] n=2	Pan *et al*.[53] n=1	Zhu *et al*.[44] n=1	0	n=8
*LIN28*	Oatley *et al*.[3] n=1	Not reported	Not reported	Not reported	0	n=1
*NANOS2*	Oatley *et al*.[3] n=1	Not reported	Not reported	Not reported	0	n=1
*CD9+*	Cai *et al*.[68] n=1	Not reported	Not reported	Kaul *et al*.[69] n=1	0	n=2
*C-KIT*	Dirami *et al*.[70] n=0	Not reported	Not reported	Heidari *et al*. [60], Heidari *et al*.[62], Dirami et al. [10] n=3	0	n=3
*CXCR4*	Giassetti *et al*.[45] n=1	Not reported	Not reported	Not reported	0	n=1
*Αintegrin*	Giassetti *et al*. [45], De Barros *et al*. [50], Giassetti *et al*. [48], Kim *et al*.[22] n=4	Not reported	Not reported	Kumar *et al*.[14] n=1	0	n=5
*CD49f*	Not reported	Not reported	Not reported	Wu *et al*. [71], Zhu *et al*. [44]	0	n=2
*NANOG*	Lee *et al*. [40] n=1	Not reported	Not reported	Not reported	0	n=1

SSC=Spermatogonial stem cells

### Transfection of SSC

Transfection involves the methodologies of introducing foreign nucleic acids into host cells with integration in the cell genome. Transfection of SSC attempts was conducted in 11/93 studies (Table-3) [22,23,24,30,34,51,52,55,56,59,72]. The SSCs have been commonly transfected using viral vectors for gene delivery across the host cell membrane into the cell cytosol. Optimization of the SSC transfection methods and efficiency using the enhanced green fluorescent protein (eGFP) was done in 7/11 studies. The purpose for using the eGFP was to optimize the transfection protocols specifically for SSC and transmission of the gene was confirmed by the presence of green fluorescence in the donor cell population under a fluorescent microscope or flow cytometry. The eGFP-transfected cells were transplanted into the recipient testis and the animal was castrated after a period of time ranging from 1 week to 8 weeks. Colonization of donor cells or donor-derived spermatogenesis was evaluated through the detection of fluorescent donor cells in seminiferous tubules of recipients [22,23,73]. If donor SSCs are transplanted into compatible recipients, donor-derived spermatogenesis is expected if the donor cells successfully colonized the seminiferous tubules [74]. Detection of eGFP expressing spermatozoa and eGFP expressing embryos after *in vitro* fertilization using transgenic semen was successful [24,75]. The findings indicated that transduced SSCs were able to colonize the recipient testis, initiate donor-derived spermatogenesis, and produce transgenic sperm. However, quantification of the percentages of transgenic donor sperm/DNA was not reported.

**Table 3:** Summary of reports on the methods of SSC transfection (n=10).

Author	Country	Species	Transfection method	Transgene	Transfection efficiency
Tajik *et al*. [30]	Iran	Bovine	Lipofectamine	GFP	37% uptake of transgene
Kim *et al*. [24]	Korea	Pig	Lentivirus vector	GFP	Detection of eGFP transgene in the donor-derived transgenic sperm and embryos after ICSI. 33% (eGFP expressing sperm was produced by the two of six recipient pigs) of the transplanted recipients produced transduced sperm containing the genetic modification
Tang *et al*. [25]	Canada	Pig	nucleofection	*Duchenne muscular dystrophy* gene construct TALENs DNA+eGFP	2.80-9% indel mutations detected. Lower survival of cells
Kim *et al*. [22]	Korea	Bovine	Lentivirus vector	e-GFP	The transduction efficiency was estimated to be 17% of donor-derived genetically modified cells which were present in the testes of recipient mice 2-3 months after xenotransplantation as evidenced by expression of eGFP
Abbasi *et al*. [23]	Iran	Goat	Lentivirus	eGFP	72% of enriched cells were positive for eGFP, transduced-enriched goat SSCs could colonize within the cells into the seminiferous tubules of germ cell-depleted recipient mice
Rodriguez- Sosa *et al*. [73]	Canada	Sheep	Lentivirus	eGFP	Donor cells expressing eGFP detected in 0.2% ST of mice at 2 months
Cai *et al*. [76]	China	Goat	Lipofectamine	siRNAs targeting EZH2 gene	EZH2 knockdown decrease cell viability
Zeng *et al*. [75]	USA	Pig	AAV vector, LV	eGFP	GFP in (20% and 5.9% of ejaculates. The percentage of ejaculates that were positive for the EGFP transgene ranged from 0% to 54.8% for recipients of AAV vector transduced germ cells (n ¼ 17) and from 0% to 25% for recipients of LV vector transduced germ cells
Song *et al*. [20]	China	Goat	Lipofectamine	p*PLZF*-IRES2-EGFP	Overexpression of *PLZF* increased SCC survival and renewal
Zeng *et al*. [77]		Goat	Nucleofection	Transgene constructs both hGH and CBGI	Genomic PCR for hGH and CBGI sequences; 31.3±12.6% of ejaculates were positive for both hGH and CBGI
Kim *et al*. [96]	Korea	Pigs	Electroporation	eGFP	Transfection efficiency (>7.5%) and higher survival rates of cells (>80%)

SSC=Spermatogonial stem cells, GFP=Green fluorescent protein, eGFP=Enhanced green fluorescent protein, EZH2=Enhancer of zeste homolog 2, PCR=Polymerase chain reaction, hGH=Human growth hormone, CBGI=Chicken beta-globin insulator

Lipofectamine transfection was carried in 3/11 studies. In the first study, the eGFP gene was transfected successfully into bovine SSC (transfection rate of 37%) [30]. In the second study, the *Enhancer of zeste homolog 2* gene was successfully knocked out in goat SSC using iRNAs against the gene [76]. In the third study, recombinant plasmid (*pPLZF-IRES2-EGFP)* and lipofectamine reagent were effectively transfected into goat SSC to overexpress *PLZF* protein. Notably, the findings concluded the achievement of desired transfection effect through the use of liposomal carriers. The other method of transfection according to the review findings was nucleofection (2/11). Nucleofection was successfully used to deliver transcription activator-like effector nucleases targeting *Duchenne muscular dystrophy* gene locus into porcine SSC nucleus [25]. Insertions and deletion mutations were detected in up to 18% of transfected cells. A similar technique was also used to deliver a transgene construct harboring the human growth hormone gene (hGH) and a chicken beta-globin insulator (CBGI) sequence in goat SSC [77]. These transfected SSCs were transplanted into recipient bucks. Genomic analysis of the recipient’s semen revealed the presence of hGH and CBGI sequences in 31.3±12.6% of ejaculates [77]. Finally, electroporation of SSC was reported in a single study, in which eGFP plasmid was introduced into porcine SSC. The cells were cultured and evaluated for green fluorescence, the transfection efficiency >7.5%, and 80% survival rates of cells (Table-3) [78].

### Recipient preparation methods in livestock species for germ cell transplantation

Successful transplantation of SSC and production of semen of donor-derived genotype would enable utilization of this technology in natural livestock breeding systems and also gene-editing platforms (Table-4) [7,11,13,23,24,26-28,59,72, 73,75,77,79-87]. From the review findings, SSC transplantation was conducted in four studies in bovine, four in goats, five in sheep, four in pigs, and one in camel. Transplantation of testicular cells into livestock recipients began in 2002 [79-81], although earlier reports had documented transplantation of livestock species SSC into mice recipient testes [11,13].

**Table 4:** Studies reporting on transplantation of SSC and donor-derived spermatogenesis in livestock.

Author	Country	Species	Testis cells	Recipient species	Colonization of transplanted cells	Donor spermatogenesis
Honaramooz *et al*. [80]	USA	Goat	Fresh	3-5 months intact germline goats	Fluorescent cells in the basement membrane of ST of recipients for 12 weeks	None
Herrid *et al*. [72]	UAE	Camel	Fresh	*DBA* germline ablated camels	Microsatellite detection of donor DNA in sperm	Not able to quantify the donor sperm percentages in the ejaculate
Shirazi *et al*. [59]	Iran	Goat	Cultured > 3 weeks	Busulfan treated mice	Labelled cells in mice seminiferous tubules	None
Rodriguez - Sosa *et al*. [73]	Canada	Sheep	Fresh	Germline intact sheep	eGFP donor cells were in the average of 0.2% of tubules after 2 months	None
Mikkola *et al*. [84]	Finland	Pig	Fresh	Busulfan treated (in feed) pigs with immotile short-tail sperm defect	Donor-derived DNA in semen	Motile sperm in recipients with immotile short-tail sperm defect
Herrid, *et al*. [27]	Australia	Bovine	Fresh	Intact germline bulls	Fluorescent labelled cells in BM of ST of recipients for 6 months	Not reported
Izadyar *et al*. [81]	Netherlands	Bovine	Fresh	Irradiated bovine	Colonization determined by *DBA* staining	(Confirmation of donor sperm reported)
Stockwell *et al*. [82]	Australia	Bovine	Fresh	Germline intact bovine	Presence of fluorescent cells in ST and spermatogenesis	Microsatellite detection of for presence of donor DNA in the ejaculate
Honaramooz *et al*. [79]	USA	Pig	Fresh	Germline intact pigs	Fluorescent labelled cells in BM of ST of recipients	None
Kaul *et al*. [26]	India	Goat	Fresh	Germline intact goats	The fluorescent cells were observed up to 12 weeks after transplantation	None
Honaramooz *et al*. [80]	USA	Goat	Fresh	Germline intact goats	Donor-derived spermatogenesis	Sperm carrying the donor-derived transgene human alpha-1 antitrypsin expression construct detected in the ejaculates
Joerg *et al*. [85]	Switzerland	Bovine	Fresh	Non-mosaic Klinefelter bovine	The donor cells were rejected	None
Oatley *et al*. [13]	USA	Bovine	Fresh, Cultured cells	Busulfan-treated mice	Fresh cells colonized ST. Cultured cells did not	None
Dobrinski *et al*. [11]	USA	Bovine	Cultured > 3 weeks	Busulfan mice	Colonization of ST basement membrane	None
Herrid *et al*. [28]	Australia	Sheep	Fresh	Irradiated sheep	Donor DNA detected in the ejaculate	Microsatellite detection of donor DNA in ejaculate
Herrid *et al*. [86]	Australia	Sheep	Fresh	Irradiated sheep	Donor DNA detected in the ejaculate	Microsatellite detection of donor DNA in ejaculate
Kim *et al*. [24]	Korea	Pig	Fresh (pLV-TH-GFP) cells	*In utero* busulfan treated pig	Colonies of transduced SSC in the recipients’ testes.	eGFP expressing ejaculates used for ICSI/IVF to produce GFP expressing embryos
Ciccarelli *et al*. [7]	USA	Boar, Goat, Bull	Fresh	*NANOS2* knocks	Donor-derived spermatogenesis	Sperm 100% donor-derived genotype
Zeng *et al*. [75]	USA	Pigs	(AAV), (LV) transduced SSC	Busulfan-treated pigs	eGFP transgene ranged from 0% to 54.8% for recipients of AAV. About 0-25% for recipients of LV transfected germ cells	Semen from AAV recipients was used for (IVF), 9.09% and 64.3% of embryos were transgenic
Rodriguez - Sosa *et al*. [73]	Canada	Sheep	Fresh	Non-treated sheep	Donor cells expressing eGFP detected in ST at 2 months	None
Abbasi *et al*. [23]	Iran	Goat	Fresh LV-EGFP transduced cells	Busulfan-treated mice	Transduced goat SSCs colonized mice seminiferous tubules	None
Oatley *et al*. [87]	USA	Bovine	2 weeks culture in testes explant	Busulfan-treated mice	Colonies of SSC in the seminiferous tubules of mice	None
Zeng *et al*. [77]	USA	Goat	Fresh transduced (human growth hormone-GH) SSC	Irradiation	Of 62 ejaculates, 63.9±17.3% were positive for hGH and 42.5±12.0% were positive for CBGI	Donor-derived spermatogenesis
Stockwell *et al*. [83]	Australia	Ram	Fresh	Irradiated ram	Donor DNA detected in the ejaculate	Low levels of donor DNA

SSC=Spermatogonial stem cells, GFP=Green fluorescent protein, eGFP=Enhanced green fluorescent protein, hGH=Human growth hormone, CBGI=Chicken beta-globin insulator, GH=Growth hormone, ICSI/IVF=Intracytoplasmic sperm injection/*In vitro* fertilization, AAV=Adeno-associated virus, IVF, LV=Lentiviral vectors, ST=Seminiferous tubules, BM=Bone marrow

The success of transplantation with resultant donor-derived spermatogenesis can only be achieved through endogenous depletion of the germ cell layer of the recipient animal. In the current review, ablation of the germline layer through irradiation was conducted in five studies, ablation through the use of chemotoxic drug busulfan carried out in three studies; use of *DBA* in one study, and *NANOS2* gene knockout (n=1) (Table-4). Busulfan germline ablated mice recipients were used in five studies for evaluation colonization of labeled SSC into the seminiferous tubules. When transplantation was done using mice recipients or germline intact livestock recipients, the donor SSCs were labeled with fluorescent markers such as Red linker dye [26] or transfected with eGFP before transplantation. The fluorescence enables the identification of donor cells or spermatozoa through fluorescent microscopy or flow cytometry. Transplantation of SSC into germline intact was conducted in earlier studies in boars, bucks, and sheep [26,27,73,74,79,82].

Exposing the testes to prescribed doses of irradiations destroys the germline layer. This method was used in several studies to prepare recipients and reported the presence of donor DNA or donor transgenes in the semen of recipients following transplantation of donor SSC [28,77,81-83]. Use of irradiated recipients was an effective method of recipient preparation although the challenges included; (i) use of high levels of irradiation would cause bone marrow depression and systemic toxicity, (ii) there was a decline in donor spermatozoa in semen ejaculates of recipients over a period and quantification of donor DNA was a problem due to low percentage in semen [82].

Treatment with the busulfan drug temporarily ablates the germline layer giving a narrow window for regeneration of donor-derived spermatogenesis after transplantation of SSC. In the three studies that prepared recipients using busulfan treatment, there were reports of derived spermatogenesis through the detection of donor DNA in semen [84] or eGFP expressing spermatozoa that were used for *in vitro* fertilization to produce eGFP expressing embryos [24,75]. Although quantification of the levels of donor DNA in the semen from the recipients was not carried out and the low levels of donor DNA were indicative of a low percentage of donor-derived spermatogenesis due to the presence of endogenous spermatogenesis. Germline ablation through treatment with *DBA* was conducted in camels in one study. Donor-derived DNA was detected in the ejaculates of *DBA* treated recipients following transplantation of the testis cells [72]. To overcome the challenges associated with temporary ablation of germline layer, recently genetically germline deficient recipients were generated [7]. The recipients were generated through knockout of *NANOS2* gene, which is responsible for germline development. Hence, the germline layer fails to develop, but somatic cell support is fully developed and functional [7,88]. Transplantation of SSC into the *NANOS2* knockout boar, bucks, and bull recipients resulted in the regeneration of complete and continuous donor-derived spermatogenesis [7] (Table-4).

## Discussion

This study compiled the literature published on *in vitro* culture systems and applications in livestock production from January 1990 to February 2021. The main focus of the review was to have an overview of the current developments in SSC culture techniques, methods of recipient preparation, and intra-testicular transplantation methodologies in livestock. The emergence of precise gene-editing technologies such as clustered regularly interspaced short palindromic repeats (CRISPR) provides an opportunity for harnessing SSC potential as a transgene carrier, for *in vitro* gene manipulation and development of transgenic animals. With the low number of SSC in the total testicular cell population, long-term *in vitro* culture of the cells is required to amplify to millions the few numbers of SSC freshly isolated, which would be adequate for gene editing and transplantation [1].

From the current review, the methodology for long-term culture is still not fully standardized and commonly carried out as it was reported only in seven studies. The long-term culture of SSCs involved either of the two strategies: First, by supplementation of culture medium with a cocktail of growth factors (GDNF, bFGF, LIF, stem cell factor, and SDF); second, by growing SSCs on a feeder cell layer or feeder cells-free culture using preconditioned serum-free media. Early studies utilized serum as one of the important components of culture medium, although published reports have shown that the presence of serum in the medium enhances the growth of somatic cells and inhibits SSC self-renewal [3,5,6,21]. Culture studies of goat SSC in media containing 10% FBS have been documented [14,15]. However, the cultured cells did not exhibit a typical morphology of germ cell clumps as described by Oatley et al. [3], Bahadorani et al. [89]. Additionally, transplantation of the cultured SSC which is a definitive test to verify the stem cell capacity of cultured SSC was not carried out. Recent studies for goat SSC have demonstrated the detrimental effects of serum on SSC self-renewal and the enhanced proliferation of somatic cells in SSC culture medium containing serum [21,29]. The long-term culture of SSC has failed mainly due to difficulties in providing all *in vitro* conditions that mimic the *in vivo* SSC niche, which has physical, mechanical, and chemical support by the surrounding somatic cells and lack of unique markers for SSC identification in culture [90]. There has been a controversy on the unique markers used to identify SSCs due to their non-specificity to SSC cell type, with some of the markers being expressed by other germ cell subtypes and even somatic cells. Expression of commonly SSC markers used in most studies: *UCHL1*, *OCT4*, *SOX2*, *KLF4*, and *THY1*) is not restricted to undifferentiated spermatogonia only [3,91]. At present, the only unequivocal measure of SSC existence within a culture dish is through transplantation into a germline ablated recipient testis to assess the capacity for the reestablishment of spermatogenesis. From the findings of this, none of the long-term culture studies conducted transplanted the cultured SSC to a recipient testis for the assessment of donor-derived spermatogenesis, hence the absence of enough evidence for long-term maintenance of SSC in their undifferentiated status. Most studies on successful long-term culture and maintenance of SSC in culture were based on findings from presumptive marker expression: *PLZF*, *LIN28*, *NANOS2*, and *GFRα1* with high similarity of the cell clump morphology to bonafide mouse cultures of SSC [3,5,92,93].

Notwithstanding, morphological characteristics of SSC germ cell clumps consisting of a cluster of cells resembling a bunch of grapes with clear cell borders have been documented, and similarity in morphology with livestock, SSCs have been reported [3,6,70,94]. This characteristic germ cell clump of SSC in culture is an indication of the existence of undifferentiated spermatogonia stem cells (not definitive measure) [94]. Regarding culture conditions, the use of feeder cells for SSC cultures is still taking the lead as compared to feeder-free culture. Cultures of SSC of STO feeders performed better than Sertoli cell feeders or other somatic cells (fibroblast) feeder cells. The growth of SSC on feeders presents a major challenge in transplantation of SSC as they may interfere with colonization in the recipient testes [3]; therefore, there has to be a way to culture these cells feeder free. Preconditioned media on feeder cells and culture of cells in laminin-coated plates have shown promise of success in the long-term maintenance of the SSC in culture [3,15]. Although the morphological characteristics and marker identification are promising findings toward standard conditions for long-term cultures of SSC in livestock species, the stem cell activity of the spermatogonial populations must be assessed by intratesticular transplantation to recipient livestock species.

Transplantation of SSC and regeneration of donor-derived spermatogenesis is the definitive proof and potential for using the SSC cultures in livestock production. Several hurdles have been experienced in translating the germ cell transplantation procedures to livestock species from rodents, where it has successfully been achieved. The difficulty has been attributed to the low number of SSC within the heterogeneous germ cell population and lack of methods to prepare germline ablated males with functional testicular somatic cell ultrastructure. Ablation of the endogenous germ cell layer avails empty niches for colonization by exogenous SSC, thus regeneration for donor-derived spermatogenesis. Methodology of intratesticular transplantation of the SSC through ultrasound-guided rete testis injection technique is well standardized. However, the success and sustainability of donor-derived spermatogenesis require a male that is permanently/genetically germline ablated but with functional somatic cell structures as in the case with the gene-edited *NANOS2* gene knockouts published by Ciccarelli *et al*. [7].

Chemical and physical methods to induce germ cell apoptosis have been used to prepare recipients. However, there is a gradual regeneration of endogenous recipient spermatogenesis, with the endogenous SSC occupying the stem cell niches, thus preventing effective colonization of the basement membrane by transplanted donor SSC. Hence, donor-derived spermatogenesis in such recipients becomes challenging to quantify.

Transplantation of livestock species SSC into mice recipients does not result in donor-derived spermatogenesis due to different genetic signaling mechanisms. However, the livestock SSCs can colonize the mice seminiferous tubules, as evidenced by immunohistochemistry experiments. Donor-derived spermatogenesis necessitates transplantation into the same species of animals as documented in pigs by Zeng *et al*. [75], Kim *et al*. [24].

The chemotoxic drug busulfan has detrimental effects on other fast-dividing cells such as bone marrow cells, which results in untargeted systemic damage affecting various organs in the recipient animals. In addition, the somatic cell population is also destroyed, thus affecting the robustness of spermatogenesis [91]. Regardless of the route of busulfan administration to recipient animals, whether done in feed or systemic administration, in all studies that used this drug, the recipients showed a resumption of endogenous spermatogenesis with the production of semen having the recipient’s genotype. However, donor-derived spermatogenesis also occurred with low levels of donor DNA irregularly detected by microsatellite markers, but the levels declined with time [24,75,84].

In addition, the use of irradiation to deplete endogenous germ cells is also cytotoxic to the somatic cell population, testicular seminiferous tubular structure, and the rest of the cells in the body, which may limit the capacity for donor SSC colonization. Testicular irradiation causes temporary depletion of endogenous germ cells [95]. *DBA*, which is a plant-based lectin with selective toxicity to bovine Type A spermatogonia also resulted in temporary germline ablation with the detection of donor DNA in recipients ejaculate, although the authors were not able to quantify the amount of donor-derived sperm in the ejaculate [72].

Notably, efforts have been made in using recipients with temporary germline ablation for transplantation with donor SSC. However, in most cases, the presence of both donor-derived and recipient endogenous spermatogenesis makes it challenging to quantify the intended donor-derived spermatogenesis conclusively. In general, methods that result in temporary germline ablation are ineffective, especially if the intended end result is to use the technology as an alternative breeding technology in livestock production systems. To utilize the potential of germ cell technology in livestock species, several researchers have suggested genetic engineering of surrogates/recipients that are genetically germline ablated but have intact testicular tubular structure and functional somatic cell support [88]. This will avail SSC niches that will be occupied by exogenous donor SSC to enable colonization basement membrane by these cells after transplantation. This would lead to 100% donor-derived spermatogenesis. From the findings in the review, SSC transplantation recipients were successfully generated through gene editing to knockout *NANOS2* gene. Complete donor-derived spermatogenesis was confirmed in the recipients [7].

Precise gene editing technologies are growing faster, and SSC is the new frontier for gene manipulation and dissemination of desirable elite genetics. The SSC populations are progenitor cells for spermatozoa, and hence, it would be easier to spread the gene of interest if gene-edited SSCs are used in transplantation. *In vitro* gene manipulation of SSC and subsequent transplantation will be helpful in the development of transgenic livestock. Successful attempts to generate transgenic animals by using SSC have been made in rodents [96]. So far, none of the studies in which SSC transfection was carried out aimed at the introduction of genes targeting disease resistance or production traits of interest, hence this research area remains unexploited. The main focus of the studies was to determine the potential transfection efficiency of different methods using the eGFP reporter gene or other transgenes in a few cases, of which transfection success was varied in all of the studies, with most of them below 10%. Although the findings are intriguing, there is still more research to be conducted in this area as the most efficient method of viral transduction of SSC using viral vectors has associated safety risks, especially if the cells are going to be transplanted into live recipients [77]. Other methods for introduction of exogenous genes into cells such as nucleofection [25] and lipofectamine transfection [30,76] have been shown to have great potential for exploitation of *in vitro* gene manipulation of SSC. Finally, prepubertal animals have been poised as the best donors for SSC since, at this age, most gonocytes have differentiated to spermatogonia, thus maximum harvesting of mitotically active SSC. At this age, the seminiferous tubules are only made up of Sertoli cells and undifferentiated spermatogonia/SSC [97]. Therefore, the population of germ cells isolated is likely to contain a high population of SSC.

## Conclusion

Great strides have been made on male germline stem cells culture and utilization of SSC cells in livestock production since the year 2000, when active SSC studies in livestock species were initiated. So far, the conditions for long-term culture of SSCs which include using a serum-free medium with a cocktail of growth factors with or without feeder cells have been optimized but there is still a need for more research to develop standardized methodology across the livestock species. Following the generation of ideal surrogate sires, transfer of SSC from donor male to recipients will allow the dissemination of superior gametes through breeding. However, more research on the performance of gene-edited surrogates in field conditions should be conducted, as well as assess natural mating and fertility capacity.

The successful performance of surrogate sires transplanted with donor SSC would enable effective utilization of this technology especially in arid and semi-arid regions of sub-Saharan Africa where the uptake of more commonly applied breeding technologies has largely been hindered by culture and lack of functional infrastructure. Despite a high proportion of livestock in sub-Saharan Africa being in the arid and semi-arid areas, research on this reproductive technology has not been carried out. Hence, the importance of developing the surrogate sire technology using livestock that is already adapted to these harsh environments for the benefit of inhabitant communities. The International Livestock Research Institute in Kenya, Africa, has embarked on a study to culture goat SSCs and transfer of the *NANOS*2 gene-editing technology to develop surrogate sires of indigenous goats. The success of this project in Africa will be a great milestone in the utilization of this new breeding technology in sub-Saharan Africa.

## Authors’ Contributions

WN: Contributed to data collection, data interpretation, and manuscript drafting. ANK: Contributed to data collection and manuscript review. JNM: Manuscript revision and Ph.D. supervisor. CT: Critical manuscript revision. SK: Project conceptual design, principal scientist of the project, and Ph.D. supervisor. All authors reviewed and approved the final manuscript.
